# How market integration impacts human disease ecology

**DOI:** 10.1093/emph/eoae026

**Published:** 2024-09-28

**Authors:** Lev Kolinski, Tyler M Barrett, Randall A Kramer, Charles L Nunn

**Affiliations:** Department of Evolutionary Anthropology, Duke University, Durham, NC, USA; Department of Evolutionary Anthropology, Duke University, Durham, NC, USA; Nicholas School of the Environment, Duke University, Durham, NC, USA; Duke Global Health Institute, Duke University, Durham, NC, USA; Department of Evolutionary Anthropology, Duke University, Durham, NC, USA; Duke Global Health Institute, Duke University, Durham, NC, USA

**Keywords:** market integration, disease ecology, subsistence, One Health, infectious disease, zoonotic disease

## Abstract

Market integration (MI), or the shift from subsistence to market-based livelihoods, profoundly influences health, yet its impacts on infectious diseases remain underexplored. Here, we synthesize the current understanding of MI and infectious disease to stimulate more research, specifically aiming to leverage concepts and tools from disease ecology and related fields to generate testable hypotheses. Embracing a One Health perspective, we examine both human-to-human and zoonotic transmission pathways in their environmental contexts to assess how MI alters infectious disease exposure and susceptibility in beneficial, detrimental and mixed ways. For human-to-human transmission, we consider how markets expand contact networks in ways that facilitate infectious disease transmission while also increasing access to hygiene products and housing materials that likely reduce infections. For zoonotic transmission, MI influences exposures to pathogens through agricultural intensification and other market-driven processes that may increase or decrease human encounters with disease reservoirs or vectors in their shared environments. We also consider how MI-driven changes in noncommunicable diseases affect immunocompetence and susceptibility to infectious disease. Throughout, we identify statistical, survey and laboratory methods from ecology and the social sciences that will advance interdisciplinary research on MI and infectious disease.

## INTRODUCTION

Market integration (MI) is the process by which people transition from subsistence to market-based livelihoods through changing patterns of *production for* and *consumption from* a market economy. While MI is often used interchangeably with words like ‘economic development’ and ‘urbanization’, it is distinct in that it can be operationalized at a household level through fine-grained measures of production and consumption (Table 1). Production includes the labor and time put into goods and services for commercial sale or subsistence and the selling of those goods and services on the market. These goods and services may include crops and prepared food, livestock, wage labor, construction and more. Consumption is the ownership or use of market-sourced goods and services, including food and durable household materials [8, 9].

**Table 1.** Processes of social and economic change

Process	Definition	Primary metrics
Culture change	Shifts in the knowledge required to function in a society, which forms the basis of beliefs, values and lifestyles [1]	Cultural consonance [1], acculturation [2] and lifestyle incongruity [3]
Economic development	Macro-level shift toward self-sustaining economic growth, changes in systems of production, increased technology, societal modernization and improved human livelihoods [4]	Human capital index; gross national income; gross domestic product; adult literacy rate; carbon dioxide emissions; life expectancy at birth [5–7]
Market integration	Household-level transition from subsistence practices to consumption from and production for a market economy, accompanied by a suite of affiliated sociocultural changes [8, 9]	Commercial goods ownership; household infrastructure scores; subsistence crop yield; input–output household diary; geographic proximity to markets; agricultural wealth scores [8–10]
Urbanization	Population shift from rural to urban environments [4]	Population size and density [11]

In this sense, MI is a departure from autarky (self-sufficiency), driven by both *push factors* away from subsistence lifestyles (e.g. internal population pressure, resource scarcity and displacement by outside groups) and *pull factors* towards the market economy (e.g. social capital, food security, desire for foreign goods and access to healthcare) [12, 13]. MI exists on a gradient of livelihood diversification, as households may maintain certain autarkic behaviors while incorporating other market-oriented behaviors to optimize both health outcomes and quality of life [9, 14]. Even if individuals within a single household have different levels of engagement with the market economy, resource sharing within a household economy would likely expose all individuals within the household to market-sourced goods, thus making households the sampling unit for MI [9, 12].

MI can have profound benefits when markets provide access to foods, services, medicines and technologies [8, 9]. However, participation in the market economy can also be costly to health when subsistence activities and diets are replaced with more sedentary, market-oriented activities and calorie-dense, market-sourced foods [15]. While the general trend is for MI to contribute to a rise in the burden of noncommunicable disease [15], a wealth of studies from evolutionary medicine—including several in this journal [16–18]—reveal that market participation has different effects on health depending on context. Among Daasanach pastoralists in Kenya, for example, the probability of having hypertension increased with greater MI, yet MI was also associated with improved food and water security [18]. Likewise, a comparative study among populations with different subsistence strategies, ranging from forager-horticulturalists to urban industrialists, demonstrated that acquired and innate immune proteins in mothers’ milk varied across most subsistence patterns [16]. As with these studies, research on the relationship between MI and human health has focused on noncommunicable diseases as well as growth and nutrition and immune function [3, 16, 18–23].

Fewer studies have investigated the connections between MI and infectious disease. Most of the existing studies have concentrated on soil-transmitted helminths (STHs), revealing complex and often contradicting impacts of MI on infection patterns [10, 24]. For example, MI-related changes in household infrastructure—such as wooden floors in place of dirt floors—reduced the prevalence of certain STH infections among the Shuar, an Indigenous forager-horticulturalist group in Ecuador [10]. Conversely, closer proximity to markets was associated with increased whipworm (*Trichuris trichiura*) infection risk among this population [10]. Thus, while certain dimensions of MI reduce infection risk, others elevate it. We lack a generalizable framework for understanding how MI affects infectious disease exposure and susceptibility. Disease ecology, which also integrates behavioral, community and population ecology, engages with pathogen exposures and considers how environmental and biological factors influence infection susceptibility [25]. It also embraces a One Health approach that considers the interconnectedness of humans, nonhuman animals (hereafter, ‘animal’) and environmental health [26, 27]. Therefore, disease ecology offers a toolkit for understanding how MI impacts infectious disease processes. In addition to ecology, research on MI and health requires engagement with the social and economic factors that shape infectious disease processes as individuals transition livelihood strategies. Thus, interdisciplinary collaboration is needed among experts in ecology, anthropology, economics, sociology, One Health, medicine and public health to advance understanding of the link between MI and infectious disease.

In the following sections, we draw on prior research in disease ecology to synthesize how MI might influence pathogen transmission through human-to-human and zoonotic pathways. We also discuss how MI-driven lifestyle changes may contribute to human susceptibility to infectious diseases by affecting immunocompetence. Throughout, we identify concepts and tools from disease ecology and other disciplines to advance research on MI and health. We also consider how local contexts shape the relationship between MI and health, which is important for developing effective policy interventions [28]. Thus, this research represents an exciting next step in evolutionary medicine and public health.

## HUMAN-TO-HUMAN TRANSMISSION PATHWAYS

**Figure 1.**
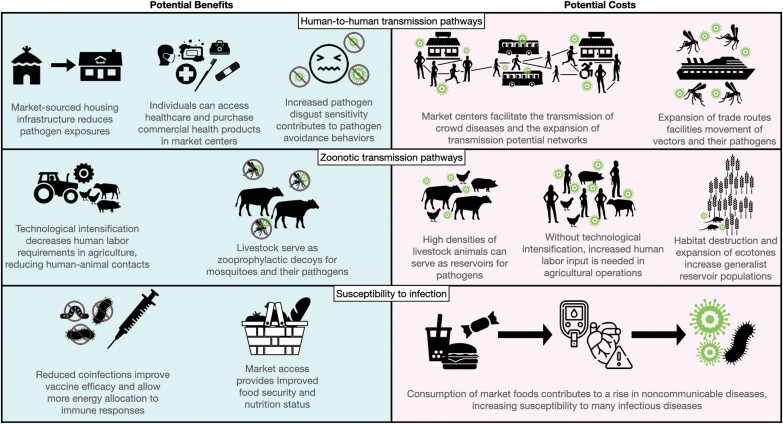
Hypothesized changes in exposure risk and susceptibility to infectious disease in the context of market integration (MI). On the left are potential benefits of MI that are expected to reduce exposure and susceptibility to infectious diseases; on the right are potential costs that are predicted to increase exposure and susceptibility to infectious diseases. For exposure risk, we consider transmission at the human–human (top section) and animal–human (middle section) interfaces

MI influences the dynamics of both close contact and non-close contact transmission (Fig. 1). Close contact transmission occurs when an uninfected individual contacts infectious stages of pathogens through proximity to an infected individual. Non-close contact transmission encompasses vector-borne transmission through arthropods like mites and mosquitoes and environmental transmission through exposure to contaminated food, water and substrates (e.g. soil and fomites). Close contact transmission is often *density-dependent*, meaning that transmission rates scale with host population density. Many forms of non-close contact transmission are *frequency-dependent*, with transmission rates depending on the rate at which an individual contacts infectious stages of pathogens. However, few infectious diseases are purely density- or frequency-dependent, and many exceptions occur. For example, sexually transmitted infections (STIs), which are spread through close contact, are better characterized as frequency- than density-dependent [25].

### Close contact transmission

MI may shape patterns of close contact transmission through its effects on population density and social networks. While linkages can exist across different subsistence populations, such as connections driven by central-place foraging patterns within hunter-gatherer societies [29], MI can change the scale of those contact patterns with implications for disease transmission. For example, the congregation of people in market centers for the purchasing and selling of commercial goods would increase density-dependent transmission. Thus, for directly transmitted pathogens, we hypothesize that greater MI increases the prevalence of ‘crowd’ diseases such as measles, COVID-19 and tuberculosis. This effect is likely to be especially strong at the early stages of MI, when population densities in market centers are low, and small increases in density scale positively with contact rates [30]. For example, in extremely dense market centers in urban areas, social contact rates likely reach a point of saturation in which further increases in population density do not affect contact rates [30]. In otherwise sparsely populated rural settings, however, the emergence of market centers characteristic of early MI can create environments of slightly higher density, and this will increase contact rates with pathogen transmission potential [30]. Although many individuals may walk to market centers at the early stages of MI, high human density and contact can occur on transportation systems used to reach markets (e.g. bush taxis common to many rural areas [31]), often in conditions of poor airflow that further increase direct contact transmission through prolonged exposure to airborne pathogens [32].

We encourage researchers to apply a network approach to investigate how MI shapes human movement and contact patterns with implications for infectious disease transmission (Table 2). Wearable GPS devices allow researchers to collect spatial data on human movement and close contacts while name-generating and travel surveys offer a quicker approach to construct networks needed to investigate transmission between market centers and rural populations [33, 34]. These networks can be combined with phylogenetic information on pathogens, medical records and survey instruments to identify transmission events and the factors that influenced them [35]. Computational experiments that simulate disease diffusion are also useful to assess the potential efficacy of public health interventions in controlling an outbreak in rural populations and can easily accommodate aspects of MI [36].

**Table 2.** Research questions, hypotheses and methods for investigating how MI shapes exposure to infectious diseases via direct and indirect contact

	Question	Hypotheses	Methods from disease ecology and other fields	Existing methods used in MI research
**Exposure via direct contact transmission**	How does MI influence disease transmission via changes to mobility and contact patterns?	MI changes the flow and scale of contact patterns among humans by creating linkages among market centers, thus increasing the scale of pathogen transmission among individuals in a population.	Social networks constructed with data from wearable GPS devices and/or name-generating surveys; models to identify whether network connections predict pathogen sharing among individuals; simulation experiments to assess the efficacy of public health interventions on infectious disease transmission.	Commercial goods ownership; household infrastructure scores; activity diaries; geographic proximity to markets.
		The scale of human–animal contact rates and the distribution of individuals involved in direct contact with animals changes as subsistence practices are replaced by market-oriented animal farming.	Human–animal networks constructed with data from wearable GPS devices and/or self-reported animal contacts; models to identify if network position predicts pathogen sharing among humans and animals; exponential random graph models to identify predictors of human-animal contact on networks.	Commercial goods ownership; household infrastructure scores; activity diaries; geographic proximity to markets; agricultural wealth scores.
	How does MI modify human behavioral immunity to pathogens at human–human and/or animal–human interfaces?	MI increases pathogen disgust sensitivity (PDS), which reduces exposure to pathogens by protecting from close contact exposures and promoting hygiene or health-seeking behaviors that prevent transmission.	Pathogen disgust questionnaires using Likert scales to assess PDS; antibody serology from dried blood spots; and effect mediation analyses.	Commercial goods ownership; household infrastructure scores.
	How does MI-driven habitat modification alter animal population dynamics with implications for zoonotic disease exposures?	Habitat modification reduces animal species diversity and increases the abundance of introduced relative to native species, leading to changes in pathogen communities.	Transect or grid methods across a gradient of habitat types to characterize animal species diversity and population composition; serology and molecular diagnostics to assess pathogen load among animals; ecological diversity indices.	Household infrastructure scores; subsistence crop yield; input–output household diary.
**Exposure via indirect contact transmission**	How do MI-driven movement and changes to the built environment impact vector metapopulation dynamics?	As communities become more interconnected by market trade, mosquito metapopulation dynamics across these populations become more similar.	Arthropod sampling for taxonomic identification and molecular diagnostics for pathogens.	Geographic proximity to markets; activity diaries.
		MI-driven household infrastructure changes reduce the abundance of arthropod vectors in the home because market-sourced housing materials are less permeable to vectors than traditional housing materials.		Household infrastructure scores.
	How do MI-driven changes to infrastructure impact pathogen diversity in soil, water and the broader environment?	MI-driven household infrastructure changes reduce the abundance of pathogens in the home when dirt floors are replaced with market-sourced flooring materials.	Point-of-sample microbial detection in water and soil samples.	Household infrastructure scores.
		Livestock farming intensification driven by MI increases the abundance of zoonotic pathogens in water systems and soil surrounding sites of livestock production.		Household infrastructure scores; agricultural wealth scores.

In addition to density-dependent transmission, frequency-dependent transmission dynamics may change with increasing MI. For example, close contact exposure to STIs may increase in settings where market participation promotes rural-to-urban migration and commercial trucking networks, both of which have been shown to increase demand for sex work and the spread of human immunodeficiency virus in Uganda [37]. Considering sex work as a livelihood strategy in the context of MI enhances understanding of STIs.

MI may also influence close contact exposure risk by modifying human behavioral immunity. Pathogen disgust sensitivity (PDS), or the degree to which the disgust emotional response drives pathogen avoidance behaviors, has been shown to increase with MI [38]. This increase occurs because individuals in more market-integrated households can afford to avoid pathogens more than individuals in less market-integrated households [38]. Specifically, market-sourced infrastructure and access to clean water and soap may determine if individuals can act on their PDS and understanding of hygiene practices [38, 39]. Therefore, individuals from more market-integrated households may be more avoidant of sources of close contact exposure (e.g. sick individuals, bodily fluids, etc.) and/or better equipped to engage in personal hygiene or health-seeking behaviors that prevent transmission (e.g. hand washing, bathing and consulting with a community health worker or medical provider).

Future studies of MI and behavioral immunity can incorporate serological methods to investigate whether MI-driven changes in PDS reduce exposure to specific pathogens. PDS can be measured in the field using pathogen disgust questionnaires, in which individuals are presented with a variety of scenarios involving potential pathogen exposure (e.g. sharing food with someone who is sick) and asked to rank how disgusted they would feel in that scenario on a Likert scale [38]. PDS is calibrated differently depending on the local context [38]; thus, we advocate for more cross-cultural studies to understand how different environments shape PDS. Coupled with metrics of MI (see metrics in Table 1) and antibody serology from dried blood spots [40], researchers can identify how MI shapes PDS and pathogen exposures through effect mediation analyses [38]. MI mediates PDS, which in turn may protect against pathogen exposures. MI may impact pathogen exposures through other mechanisms, too. With mediation analyses, researchers can determine the relative effect of PDS on pathogen exposures compared to the effects of other, unmeasured MI-related variables [38, 41].

### Non-close contact transmission

Non-close contact pathogen transmission may also be impacted by MI-driven changes in vector distribution, household infrastructure and hygiene products.

MI leads to the expansion and intensification of trade routes and individual travel, and this movement of humans and goods facilitates the accidental movement of arthropod vectors. For example, the use of river boats for market trade in the Peruvian Amazon has contributed to the spread of dengue fever when boats unknowingly carry *Aedes aegypti* mosquitoes, a key vector of dengue and other flaviviruses, to new locations [42]. Likewise, human movement on these trade routes can introduce arboviruses to new populations of mosquito vectors [43]. Conversely, market-sourced housing materials may buffer vector-borne disease risk by reducing the ability of vectors to enter homes. Market-sourced housing infrastructure is associated with a decreased risk of malaria and Chagas disease when compared to traditional housing infrastructure because market-sourced walls (e.g. cement instead of plant materials), screened windows and durable flooring materials (e.g. wood instead of dirt) discourage mosquitoes and kissing bugs (*Triatoma dimidiata*) from entering buildings [44, 45].

Thus, MI-driven changes in both production and consumption activities may be key to understanding changing exposure patterns to arboviruses and other arthropod-borne pathogens. Longitudinal mosquito population monitoring—both to determine taxonomic composition and to detect pathogens—offers researchers a tool to identify how MI-driven changes in human movement and the built environment shape vector metapopulation dynamics with potential for pathogen transmission. For example, we may expect that as markets increase connectivity among communities, mosquito populations and arbovirus prevalences across those communities become similar [43].

Market-driven changes in household infrastructure, such as the integration of plumbing and sewage systems, will likely also reduce exposure to certain water-borne pathogens, such as *Vibrio cholerae* [46]. Access to electricity, high-quality soap and clean hot water may mitigate environmental transmission via food and other fomites [38, 39]. For example, access to refrigeration, easy ways to heat food, and knowledge of best food-handling practices (e.g. washing produce with clean water prior to consumption) reduce foodborne pathogen transmission [39]. Along with human biological sampling in studies of MI, researchers should consider soil, water and other environmental sampling to screen for pathogens with non-close contact transmission potential. With these environmental pathogen data, researchers can assess how MI affects the built environment (e.g. through changes to household infrastructure) and how these changes impact the prevalence of pathogens within an environmental context (e.g. contamination of a household’s drinking water). Point-of-sample microbial detection techniques may be particularly helpful to identify environmentally transmitted pathogens in resource-limited settings [47].

## ZOONOTIC TRANSMISSION PATHWAYS

Approximately 60% of emerging infectious diseases are zoonotic in origin, and most pandemic-causing pathogens originate in animals [48]. Therefore, from public health and One Health perspectives, it is important to understand how MI affects zoonotic disease transmission at the animal-human interface. As with human-to-human transmission, zoonotic transmission can occur via close contact with animal hosts or indirectly via contact with environmental substrates or vectors that transmit pathogens from animals to humans.

### Close contact transmission

Pastoralists who rely on their herds for subsistence and foragers who frequently hunt and consume wildlife may encounter zoonotic pathogens when provisioning their own food [26]. These human-animal contact patterns may change when market demands for agricultural and livestock products lead to farming intensification and land-use change.

In addition to subsistence-driven human–animal–pathogen encounters, intensified traditional farming practices without substantial increases in technology, such as intensification at early stages of MI, may increase rates of encounters because increased human labor input is typically used to increase yield for market sale [49]. This contrasts with mechanized farming practices characteristic of technologically intensified market production, which may reduce human labor requirements [49, 50]. Close contact exposure risk is greatest for individuals who have sustained contact with live animals and their blood, such as slaughterhouse workers [51]. Previous research found that commercial livestock farm workers exhibit increased seroprevalence of antibodies against influenza viruses (H1, H5 and H7 virus types) [50]. Individuals who are not directly involved in commercial agriculture or livestock production may still be exposed to zoonotic pathogens as consumers if domestic animals are sold and/or slaughtered at markets. High densities of animal hosts within commercial farming operations may also increase density-dependent transmission of pathogens within animal populations [49, 50]. This higher pathogen prevalence in animals increases the risk of zoonotic transmission to humans.

The expansion of commercial agriculture into previously undisturbed ecosystems creates new ecotones, or transition zones between two or more bordering habitat types, with implications for zoonotic transmission [52]. MI-driven agriculture intensification at the local level is more likely to result in the expansion of agricultural fields than technology driven intensification of existing commercial fields [53]. Generalist reservoir host species, such as white-footed mice (*Peromyscus leucopus*, the principal reservoir host of Lyme disease in many systems), often congregate in high densities within ecotones that are also used by people, thus increasing human exposure to pathogens [52, 54].

Conversely, land-use changes driven by MI may decrease or eliminate pathogens when agriculture intensification creates unsuitable habitats for wild animal disease reservoirs. If pathogens of zoonotic potential or their vectors primarily exist in intact habitats, then the conversion of those habitats into agricultural lands would eliminate pathogen risks [55]. Coupled with land-use change and elimination of some pathogens is the potential loss of ecosystem services, along with the emergence of new pathogen risks associated with human density and activity. Existing literature on agriculture intensification points to a complex relationship between land-use changes and zoonotic disease emergence [50].

Serological and molecular screening of humans and animals is crucial for understanding risks associated with MI and agricultural environments. With appropriate survey instruments that assess animal contacts and market production and consumption behaviors across a gradient of MI, these infection data can be linked to the effects of MI. Ecological metrics of species diversity within and around agricultural fields and farming operations [56] provide important insights into how MI impacts community ecology and infectious disease risks. We would expect to observe greater animal species diversity and more native animals in subsistence-oriented environments compared to market-oriented environments, as native species are typically competitively displaced by introduced animals and driven away by anthropogenic habitat modification [50, 57]. As animal ecological communities change, so too will pathogen communities, with implications for human and animal health [49, 50].

Network methods are again useful for investigating changing human-animal interactions in the context of MI. The scale of human–animal contact rates and the distribution of individuals involved in direct contact with animals will likely change as subsistence practices are replaced by market-oriented animal farming [50]. Self-reported animal contact survey data offers another approach to construct human–animal contact networks. More detailed but resource-intensive contact data can be collected by deploying wearable GPS devices on both humans and their domesticated animals. These data are useful for constructing quantitative estimates of human-animal spatial overlap, which can be represented as networks. Statistical methods such as exponential random graph models can be used to investigate the specific MI-driven lifestyle changes that increase the odds of associations between humans and a given animal species on these networks.

### Non-close contact transmission

Zoonotic disease transmission also occurs through vector and environmental transmission. Production of agricultural and animal products for market sale may create new opportunities for zoonotic pathogens to enter environmental and disease vector systems, bridging animals and their pathogens to human populations even when people are otherwise unconnected with market-oriented agricultural production.

Intensified irrigation systems for commercial farming operations may impact vector ecology, and therefore increase zoonotic disease transmission, because dams and irrigation canals can support high densities of pathogen-spreading mosquitoes [50]. This effect has been observed in outbreaks of Rift Valley Fever Virus (RVFV) in Kenya, where flooding for crop irrigation increased *Aedes* and *Culex spp.* mosquito density and subsequent RVFV transmission among cattle and humans [58]. However, reductions in vector transmission may occur when habitat fragmentation related to commercial farming reduces a vector species’ habitat, as has been observed in eastern Zambia with Tsetse flies (*Glossina morsitans morsitans*), a vector of human African trypanosomiasis [50, 59]

In the complex matrix of infectious disease transmission, an MI-related change may increase one component of zoonotic disease risk for humans while decreasing other components of such risk. For example, MI-driven increases in domesticated animal production and sale may increase exposure to some zoonotic infectious diseases, such as enteric pathogens [50]. However, livestock may decrease exposure to mosquito vectors if the domesticated animals draw mosquitoes away from humans and towards themselves, reducing the human biting rate. Known as zooprophylaxis, this phenomenon has been observed in a nationally representative study in the Democratic Republic of Congo, which reported a negative association between cattle ownership and *Plasmodium falciparum* (malaria) infection among study participants [60].

Water may be a key driver of environmental transmission of zoonotic pathogens in communities undergoing MI, especially at the early stages of water and sewage system development. For example, water contamination from commercial agriculture operations has been linked to outbreaks of zoonotic pathogens such as *Giardia* and *Cryptosporidium* in several countries [61]. Further, when synthetic fertilizers for farming enter water systems through runoff contamination, an increase in aquatic vegetation density within those systems can increase waterborne transmission of zoonotic pathogens such as *Schistosoma spp.*, as freshwater snails and other animal hosts thrive in such environments [62]. In this system, market consumption of agricultural inputs (purchasing synthetic fertilizer) is directly linked to market production (crop farming), and together, this market consumption and production promote waterborne transmission of zoonotic pathogens. Broadly, research should consider how commercial inputs for market production impact not just human lives, but also surrounding water systems and the environment.

Traditional housing infrastructure, and specifically dirt floors, may increase the risk of infections with zoonotic pathogens such as hantavirus and *Leptospira* via indirect transmission [63, 64]. Depending on environmental conditions and pathogen strain, hantavirus and *Leptospira* bacteria—both of which are commonly carried in rodent reservoirs and spillover to human populations—can persist in the dirt for prolonged lengths of time [65, 66], which creates more opportunities for transmission of infectious stages. The replacement of dirt floors with purchased flooring materials, such as wood, cement and tile, may therefore reduce opportunities for environmental pathogen exposure within the home. In addition, just as market-sourced household materials may protect against malaria and Chagas transmission among humans, more market-integrated households may be at reduced risk of arboviruses and other mosquito- or tick-transmitted zoonotic pathogens if arthropods have less access to the home [44, 45]. Household infrastructure assessments are standard to studies of MI (e.g. [10],), but the specific impacts of market-sourced building materials in the context of MI on many infectious disease systems are poorly understood.

## SUSCEPTIBILITY TO INFECTION

Disease ecology considers both exposure and susceptibility to infectious disease, where susceptibility refers to a complex set of host responses to pathogens involving resistance (i.e. killing or disarming a pathogen) and tolerance (i.e. coping with a pathogen to avoid severe illness). In this context, MI-driven lifestyle changes alter susceptibility to infectious diseases by modifying human immunocompetence.

Increased MI, and its association with dietary and activity changes (e.g. greater consumption of market foods and decreased participation in subsistence activities like hunting), has been implicated to varying extents in increased obesity, hypertension, dyslipidemia and/or impaired fasting glucose in studies among populations globally [20, 22, 23, 38]. Collectively, these risk factors for metabolic syndrome may contribute to greater burdens of Type 2 diabetes (T2D) and cardiovascular disease (CVD) [22], both of which are known to increase susceptibility to infectious disease. For example, T2D influences multiple pathways of immune dysfunction that can promote infection susceptibility via reduced resistance mechanisms: dampened neutrophil function, reduced T lymphocyte response, and altered inflammatory cytokine secretion, and by causing humoral immunity disorders and increased oxidative stress [67].

CVD also dampens the immune response to pathogen exposure. *In vitro* stimulation of monocyte-derived macrophages from patients with coronary artery disease, as well as *in vivo* stimulation in rodent models, indicate that CVD leads to diminished T-cell response to viral stimuli [68]. These examples of immune dysfunction may explain why T2D, CVD and obesity have also been linked to increased rates of severe illness (i.e. decreased tolerance) in cases of infection with viruses, helminths, fungi and other pathogens [69].

Through this lens, we can thus view the MI-driven rise in noncommunicable diseases as an example of evolutionary mismatch. Genetic studies of populations undergoing MI are elucidating relevant genotype-by-environment interactions, but more research is needed on their downstream effects on infectious disease risk in these populations [15, 70].

In addition to general immune dysfunction, MI-related noncommunicable diseases may increase susceptibility to specific pathogens. For example, obesity is associated with reduced resistance and tolerance to infection with SARS-CoV-2, potentially due to increased expression of the angiotensin-converting enzyme 2 (ACE2) gene in visceral and subcutaneous adipose tissue. SARS-CoV-2 binds to ACE2 receptors to infect host cells; thus, a greater number of ACE2 receptors in obese individuals may create more opportunities for infection with SARS-CoV-2 [71].

MI may reduce susceptibility to infectious diseases if market access improves food security and hygiene conditions. While obesity may increase one’s susceptibility to many infectious diseases, some body fat achieved through MI may decrease susceptibility by providing energy stores to launch effective immune responses to pathogens [15, 62]. For example, children with greater levels of body fat in the Ecuadorian Amazon avoid growth stunting related to immune activation, unlike children without available fat energy reserves [72]. Market access creates opportunities to consume energy-dense, market-sourced foods and this improved caloric balance may help fuel immune response without trade-offs in energy investment in other biological processes such as growth and anabolism.

Likewise, MI may reduce susceptibility to infectious diseases by reducing the burden of coinfections. For example, STH infections that are common in less market-integrated settings may increase susceptibility to other pathogens by reducing vaccine efficacy, draining energy resources and downregulating immune response to other pathogens [10, 38, 73, 74]. Therefore, MI-driven household infrastructure changes that reduce STH prevalence may also decrease susceptibility to other infections by increasing immunocompetence.

Comparative studies of populations undergoing MI in different settings are necessary to determine the distinct consumption and production mechanisms of MI that drive changes in chronic disease and susceptibility to infectious diseases. In a comparative study of peri-urban and forager-horticulturalist Shuar children, for example, peri-urban children exhibited lower total immunoglobulin (IgG and IgE) concentrations than their less market-integrated counterparts [23]. IgG is key to developing immunological memory following pathogen exposures, while IgE is generally produced in response to macroparasite infections and allergic reactions [40]. However, another study of MI among Indigenous Siberians showed that cell-mediated immune response (measured as Epstein–Barr virus antibody concentrations) was negatively associated with both subsistence and market-integrated material lifestyle scores, indicating that MI alone could not explain variation in immune function [3].

**Figure 2.**
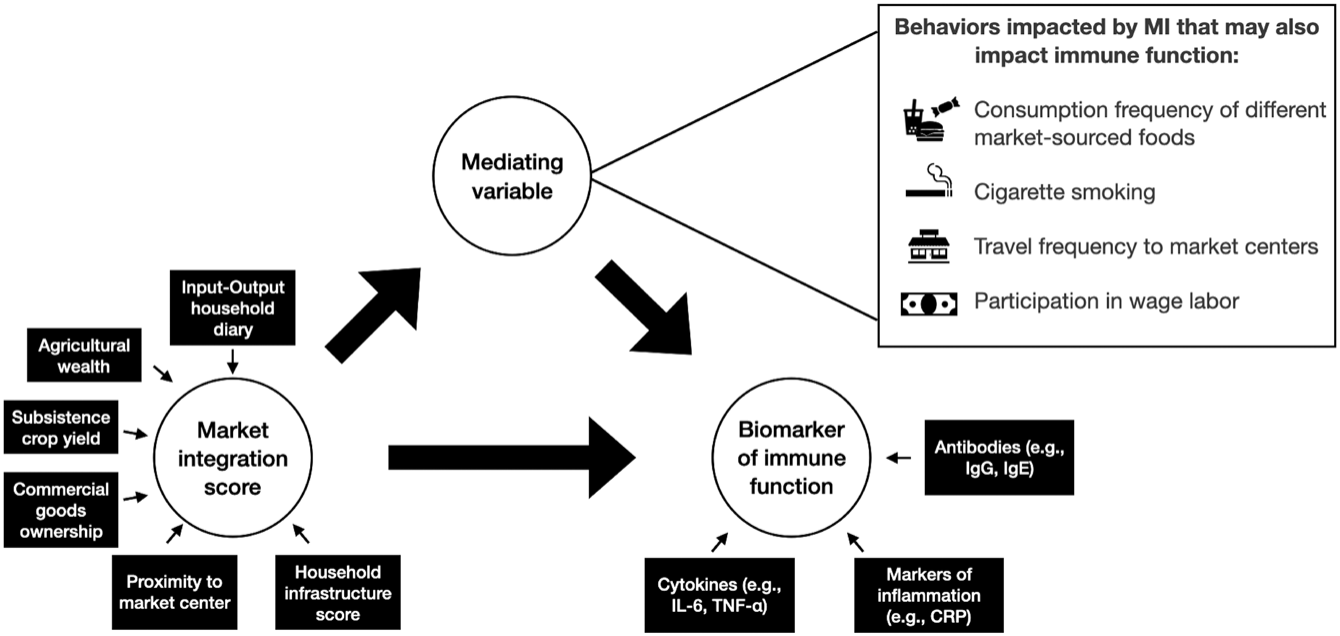
Market integration may impact immune function directly or through an intermediate mechanism. This example directed acyclic graph identifies the causal pathways linking MI to biomarkers of immune function, a measure of susceptibility to disease. MI can be assessed at the household level through measures of household infrastructure, commercial goods ownership and other metrics of market production and consumption (see Table 1). MI may directly affect immune function, or an intermediate variable along this causal pathway may affect immune function. Mediating behaviors to consider include consumption frequency of different market-sourced foods, tobacco usage and travel patterns to markets. These potential mediators are generally not used to measure MI; however, they are likely impacted by MI and may influence susceptibility. Figure adapted from Lea et al. (2020)

More population-specific research is needed to understand the mechanisms of MI that alter markers of immune function and their impacts on susceptibility to infectious diseases. Further, with longitudinal survey data, biomarkers of immune activation (see [40]), and vital health monitoring, it may be possible to capture MI-driven changes in immune function, health and disease susceptibility in real-time. Directed acyclic graphs (DAGs) and mediation analyses (with adequate sample sizes) can be useful tools in this observational research to infer the specific mechanisms of MI that cause changes in human health and infection susceptibility [41, 75]. Unlike traditional analysis methods, DAGs and mediation analyses allow researchers to incorporate prior knowledge about biological and social systems and infer causal effects of given variables [41, 75]. In research investigating how MI impacts susceptibility to infectious diseases, DAGs and mediation analyses provide researchers tools to identify the specific mechanisms of MI, related to production and/or consumption variables, that impact a given biomarker of immune function (Fig. 2).

## CONCLUSIONS AND FUTURE DIRECTIONS

The transition from subsistence to market-based livelihoods transforms human health, but its impact on infectious disease processes remains underexplored. Here, we provided a synthesis and framework that would close this research gap by leveraging concepts and tools from disease ecology to study how MI affects infectious disease exposure and susceptibility. Consistent with previous research demonstrating the positive and negative consequences of MI for human health, we described pathways by which MI may both increase and reduce infectious disease risk by modifying patterns of market production and consumption. We are left with several key takeaways and future directions.

First, it can be challenging to establish directionality when studying the relationship between livelihood transitions and human health. In the economics literature on country-level development, this problem is referred to as endogeneity: economic development is both a cause and a consequence of population health. MI is distinct in that it can be measured at the household level; however, the problem of endogeneity still may exist at this scale. Here, we aimed to present a synthesis that introduces interdisciplinary concepts and tools from disease ecology and related fields for researchers of MI. Methods from health economics may also be helpful for enriching the study of MI and infectious diseases. For example, structural equation modeling techniques such as simultaneous equations and coupled disease-economic models may allow researchers to approximate the relative effects of MI and infectious disease outcomes on each other. We encourage future work that integrates econometric methods with the methods proposed here [76, 77].

Second, MI is a human process with consequences for human health, and it also has health implications for other animals. One Health perspectives are therefore crucial for advancing this field. Although we considered disease exposure at the animal-human interface, we chose to focus on unidirectional zoonotic transmission (i.e. from animal to human). However, many infectious diseases are transmitted via cross-species transmission from humans to wildlife and domesticated animals [78]. For example, pigs (*Sus. spp.*) are susceptible to many of the same influenza viruses that infect humans [78]; thus, commercial pig farming operations for a market economy may increase cross-species transmission risks for both humans and pigs, including wild relatives of domestic pigs. We urge future MI research to consider this potential for human-to-animal transmission within a comprehensive One Health perspective.

Third, researchers should consider humans as endogenous actors within the disease and environmental systems that they occupy. Humans have changed subsistence strategies with implications for the environment throughout our evolutionary history. Moralizing language about the ‘destructive’ nature of habitat modification by populations undergoing contemporary MI fails to acknowledge the role of many populations in high-income countries in driving habitat degradation over hundreds of years of industrialization and population growth. Instead, we encourage an approach to ‘meet people where they are’ and to understand human behavior as a natural force within disease ecology [26].

MI is a population-specific process. MI of forager-horticulturalists such as the Tsimane of Bolivia [24] will look different than that of Daasanach semi-nomadic pastoralists in Kenya [18]. Thus, a research framework that prioritizes the health and economic concerns of those undergoing MI and centers local knowledge is key when planning future research on MI and infectious diseases. We encourage a community-based participatory research approach [79] in which researchers align and work with stakeholders to learn their principal health concerns and then design studies to address those concerns. For example, in settings where individuals are most concerned with malaria, we would advise centering infectious disease research on malaria. Researchers should also consider how traditional health knowledge may impact infectious disease processes. Traditional knowledge may often prevent infectious disease exposure and reduce susceptibility (e.g. taboos about food may protect from exposure to foodborne pathogens) [38]. Researchers can use a biocultural approach to consider how market consumption and production coexist with and affect this traditional knowledge through processes of acculturation (see [8]). With human serological methods and assessments of health knowledge, it would be possible to assess how MI mediates health knowledge with potential implications for infectious disease exposures.

By incorporating concepts and tools from disease ecology, the MI research community is well-positioned to provide a richer analysis of local contexts and to further understanding of disease transmission as a biosocial process [28].
